# Interstitial granulomatous dermatitis secondary to microscopic polyangiitis

**DOI:** 10.1016/j.jdcr.2025.10.050

**Published:** 2025-11-03

**Authors:** Sabrina F. Schundler, Elise K. Brunsgaard, Vijaya B. Reddy, Marie D. Lafeir

**Affiliations:** a Department of Dermatology, Rush University Medical Center, Chicago, Illinois; b Department of Pathology, Rush University Medical Center, Chicago, Illinois

**Keywords:** ANCA-associated vasculitis, annular rash, interstitial granulomatous dermatitis, leukocytoclastic vasculitis, microscopic polyangiitis, reactive granulomatous dermatitis

## Introduction

Microscopic polyangiitis (MPA) is a rare form of vasculitis associated with anti-neutrophil cytoplasmic antibodies (ANCA), most commonly myeloperoxidase (MPO)-ANCA, and can present with multi-organ involvement, most frequently affecting the kidneys, lungs, and skin.^1^ Cutaneous manifestations of ANCA vasculitides are diverse and have been reported to include palpable purpura, livedo reticularis, nodules, ulcers with necrosis, bullae, erythema elevatum diutinum-like plaques, and urticaria.^1^ Recognizing these skin presentations is crucial, as they can be the initial or a concurrent sign of systemic vasculitis, aiding in early diagnosis and management of the disease. However, other dermatoses, such as interstitial granulomatous dermatitis (IGD), have rarely been described in the setting of MPA. In this report, we present a case of IGD secondary to MPA and further explore the term reactive granulomatous dermatitis.

## Case report

**Fig 1:**
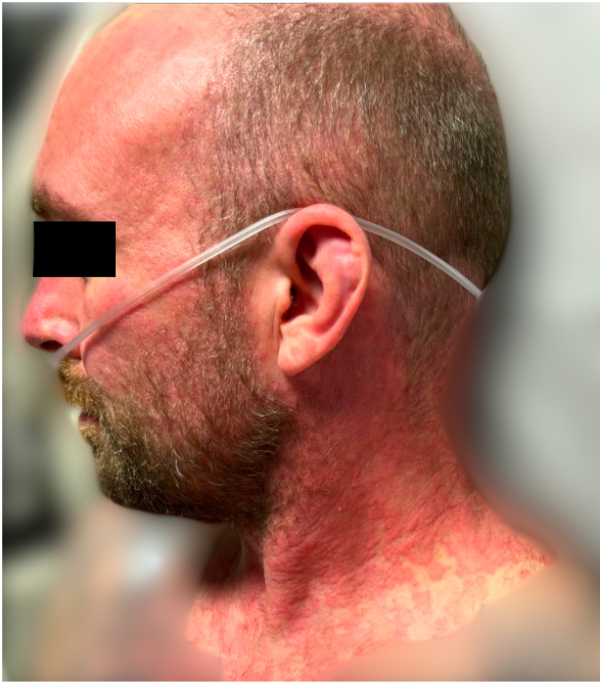
Diffuse erythematous, annular plaques involving the face and neck.

**Fig 2:**
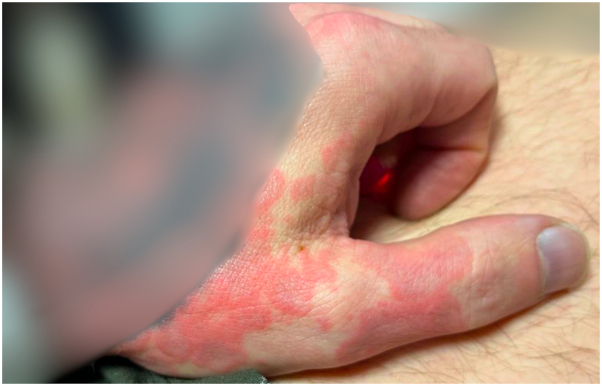
Erythematous, annular, targetoid macules involving the dorsal surface of the left hand.

The patient is a 40-year-old male who presented to the emergency room with 10 days of cough, intermittent fevers, and shortness of breath with progressively worsening hemoptysis. One week prior, he had presented to an outside hospital where he was treated for community-acquired pneumonia with ceftriaxone and doxycycline. While admitted, he was noted to have diffuse, annular plaques involving the face, neck, upper back, upper chest, forearms, and hands (Figs 1 and 2). He endorsed a burning sensation associated with the rash, but he denied any significant pain or pruritus. His past medical history was significant for immune thrombocytopenic purpura and ankylosing spondylitis. One month prior to presentation, he began adalimumab for treatment of ankylosing spondylitis and had received 2 doses of the medication.

**Fig 3:**
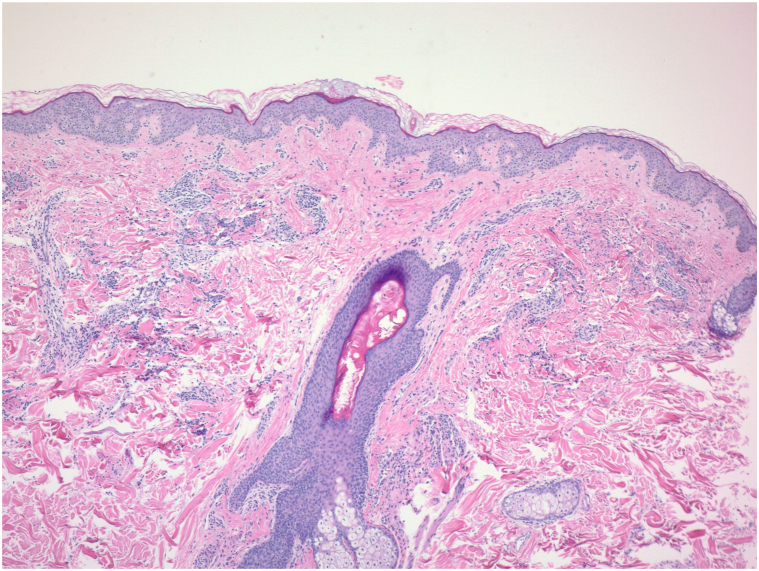
Hematoxylin and eosin, original magnification ×40: Histopathologic examination of a punch biopsy of skin from the shoulder shows mixed superficial perivascular and interstitial infiltrate.

Laboratory evaluation revealed an elevated CRP. Indirect immunofluorescence staining demonstrated a p-ANCA pattern and enzyme immunoassay confirmed the presence of MPO antibodies. Bronchoscopy showed evidence of diffuse alveolar hemorrhage. A punch biopsy of skin from his shoulder was performed which showed perivascular and interstitial infiltrate of lymphocytes, monocytes, and scattered eosinophils (Fig 3). Immunohistochemical stains highlighted CD68-positive interstitial histiocytes. This histologic pattern was consistent with interstitial granulomatous dermatitis. Additionally, he underwent renal biopsy which demonstrated evidence of pauci-immune glomerulonephritis. Based on his clinical history, laboratory workup, and these histologic findings, the patient was diagnosed with MPA.

The patient was started on intravenous methylprednisolone 1 gram daily for 3 days and triamcinolone 0.1% ointment was applied to the affected areas which resulted in marked improvement in symptoms. The patient was transitioned to oral prednisone 60 mg daily on discharge. Two weeks later, the annular plaques had resolved with only mild residual erythema. For management of his MPA, rheumatology treated the patient with rituximab, 1 gram every 2 weeks for 2 doses, with the plan to repeat this dosing every 6 months. For 4 months, the patient was on methotrexate 10 mg weekly which was discontinued due to transaminitis. He was transitioned to azathioprine and titrated to 250 mg daily. His prednisone was gradually tapered and ultimately discontinued, completing a total of 13 months of steroid therapy following diagnosis.

## Discussion

Our case of IGD represents a rare cutaneous presentation of MPA, adding to the known dermatologic manifestations of ANCA-associated vasculitis. Histologically, IGD can be identified by the visualization of inflammatory infiltrate, commonly CD68+ epithelioid histiocytes, scattered throughout the dermis in varying densities, often with high concentrations around degenerated collagen.^2^ Rarely, eosinophils and neutrophils may also be visualized, however vasculitis is generally not seen.^3^ Histiocytes may aggregate into small granulomas and cluster around abnormal collagen, creating clefts known as the “floating sign,” which has been observed in approximately two-thirds of cases.^3^

IGD is most commonly observed in the setting of an underlying systemic disease such as rheumatoid arthritis, systemic lupus erythematosus, and solid organ malignancies.^2^ A previously reported case of palisaded neutrophilic dermatitis (PNGD) in the setting of ankylosing spondylitis described skin lesions that developed 2 months after joint symptoms and improved with treatment of the underlying disease.^4^ In contrast, our patient had joint symptoms for approximately 2 years before hospitalization and skin lesion onset and, now over a year after resolution of IGD and well-controlled MPA, continues to be managed for ongoing ankylosing spondylitis symptoms. A drug-induced mechanism has been proposed given there have been rare cases of TNF alpha inhibitor-induced IGD.^5^ The patient last received adalimumab 2 weeks before presentation, and lesions cleared within 2 weeks of initiating MPA treatment. Reported cases of TNF alpha inhibitor-induced IGD describe onset within 1 month to over a year after drug initiation and resolution 1 to 18 months after discontinuation.^5^ Based on simultaneous onset of cutaneous symptoms with development of cough, intermittent fevers, shortness of breath, and hemoptysis, as well as resolution with treatment for ANCA vasculitis, we favor the IGD was associated with his presentation of underlying ANCA vasculitis rather than secondary to ankylosing spondylitis or TNF alpha inhibition.

The exact association between ANCA-associated vasculitis and IGD has not been well-investigated. ANCA-associated vasculitides are all characterized by inflammation of small to medium-sized blood vessels, where neutrophils play a key role in mediating tissue damage, which may suggest a common inflammatory pathway between IGD and ANCA-associated vasculitis.^6^ In granulomatosis with polyangiitis (GPA), a form of ANCA-associated vasculitis, granulomatous inflammation is a defining feature, resembling the granuloma formation observed in IGD.^7^ IGD has been reported in association with GPA, suggesting a potential shared underlying pathophysiology.^8^

The term reactive granulomatous dermatitis has been proposed as an umbrella term to encompass IGD, interstitial granulomatous drug reaction, and PNGD to reflect their shared characteristics as a reaction pattern associated with underlying systemic disease.^2^ A pathologic study of IGD versus PNGD found overlapping interstitial and palisaded inflammatory patterns in over 90% of cases, favoring they belong on the same clinicopathologic spectrum.^9^ Additionally, there was no correlation between the predominant infiltrate pattern and the tissue neutrophilia.^9^

This case emphasizes the importance of recognizing reactive granulomatous dermatitis as a potential indicator of underlying systemic disease, particularly MPA. Our patient's presentation with diffuse annular plaques and targetoid macules with histopathologic findings consistent with IGD adds to the diversity of clinical presentations of vasculitis-associated dermatologic findings.

## Conflicts of interest

None disclosed.
